# Implementing community-based human papillomavirus self-sampling with SMS text follow-up for cervical cancer screening in rural, southwestern Uganda

**DOI:** 10.7189/jogh.11.04036

**Published:** 2021-12-25

**Authors:** Naima T Joseph, Alexcer Namuli, Bernard Kakuhikire, Charles Baguma, Mercy Juliet, Patience Ayebare, Phionah Ahereza, Alexander C Tsai, Mark J Siedner, Thomas R Randall, Joseph Ngonzi, Adeline A Boatin

**Affiliations:** 1 Department of Obstetrics and Gynecology, Beth Isreal Deaconness Medical Center, Boston, MA, USA; 2 Harvard Medical School, Boston, MA, USA; 3 Department of Obstetrics & Gynecology, Mbarara Regional Referral Hospital, Mbarara, Uganda; 4 Mbarara University of Science and Technology, Mbarara, Uganda; 5 Center for Global Health, Massachusetts General Hospital, Boston, MA, USA; 6 Division of Infectious Disease, Department of Medicine, Massachusetts General Hospital, Boston, MA, USA; 7 Department of Obstetrics & Gynecology, Massachusetts General Hospital, Boston, MA, USA

## Abstract

**Background:**

Self-collected HPV screening may improve cervical cancer screening coverage in low resource countries, yet data guiding implementation and follow-up of abnormal results are sparse.

**Methods:**

This is a prospective cohort implementation study of HPV self-testing program in Mbarara, Uganda with mobile phones to facilitate result notification and referral for treatment at a regional hospital. The effectiveness of the interventions was analyzed using Proctor’s model of implementation. Women were interviewed following screening and at 6 months to assess acceptability and barriers to follow-up. Data were analyzed using descriptive statistics.

**Results:**

159 of 194 (82%) of eligible women underwent HPV self-sampling; of these, 27 (17%) returned positive for high-risk HPV subtypes. We sent SMS messages providing test results and follow-up instructions to all participants. Seventeen (63%) hrHPV-positive participants reported receiving SMS text instructions for follow-up, of whom 6 (35%) presented for follow-up. The most common reasons for not returning were: lack of transportation (n = 11), disbelief of results (n = 5), lack of childcare (n = 4), and lack of symptoms (n = 3). Confidence in test results was higher for self-screening compared to VIA (Likert score 4.8 vs 4.4, *P* = 0.001).

**Conclusions:**

Despite the use of SMS text-based referrals, only one-third of women presented for clinical follow-up after abnormal HPV testing.

Cervical cancer is a leading cause of preventable cancer morbidity and mortality, contributing approximately 250 000 deaths in low- and middle-income countries (LMICs) annually [1]. The 90-79-90 campaign launched by the World Health Organization (WHO) aims to eliminate cervical cancer as a public health burden by vaccinating 90% of the eligible population, screening 70% of women and treating 90% of women in need by 2030 [2]. Despite the availability of effective screening methods for premalignant lesions (cytology, visual inspection with acetic acid (VIA), and human papillomavirus (HPV) testing), only 10%-20% of eligible women in LMICs are appropriately screened – largely due to barriers of patient access, technical expertise, laboratory capacity, and scalability [3-6]. Low-cost, easy to use, and scalable techniques for screening are needed to meet targets for the next decade.

Recognition of the strong causal relationship between persistent cervical infection with high-risk human papillomavirus (hrHPV) subtypes and cervical cancer has led to a proliferation of HPV molecular assays. Compared with VIA and cytology, hrHPV DNA testing has a higher sensitivity and negative predictive value for precancerous lesions, less inter-observer variability, a more favorable cost-effectiveness profile, and remains efficient in settings with high HPV vaccine penetration [7-10]. Importantly, hrHPV testing allows for self-collection of samples, which is accurate, feasible, and averts the logistical and human resource challenges associated with clinician-based screening [10-12]. For these reasons, hrHPV testing has been recommended by the WHO as a preferred screening strategy when feasible [3,13].

Where cultural and program barriers limit women’s access to clinician-based cervical cancer screening, home- or community-based self-sampling methods can increase participation among women who are difficult to reach through conventional means [14]. However, there are sparse guidelines on the implementation of hrHPV self-sampling in LMICs and limited data on the best methods to improve linkage to care and follow-up of HPV-positive women. Mobile phone text messages can be used to improve follow-up and participation in HIV in LMICs, but there are limited data on the role of text messaging to improve linkage to care after cervical cancer screening [15-17]. To address this knowledge gap, we leveraged a prospective cohort study with integrated community health fairs to pilot hrHPV self-testing in a community setting and implement mobile phone-based results notification to facilitate follow-up treatment at a regional cervical cancer prevention clinic.

## METHODS

The parent study is a population-based sociocentric social network cohort in a rural administrative sub-unit of Rwampara District in southwestern Uganda, whose primary objective is to characterize the social network context of HIV stigma and understand its impacts on HIV care. Community feedback on study procedures is both formally and informally sought on a regular basis, through a community advisory board and multiple community sensitization meetings [18]. In response to community feedback, we incorporated cervical cancer screening (using VIA) into the study in 2017, leveraging the ongoing community health fair infrastructure to perform this pilot study of community-based hrHPV screening.

**Figure 1:**
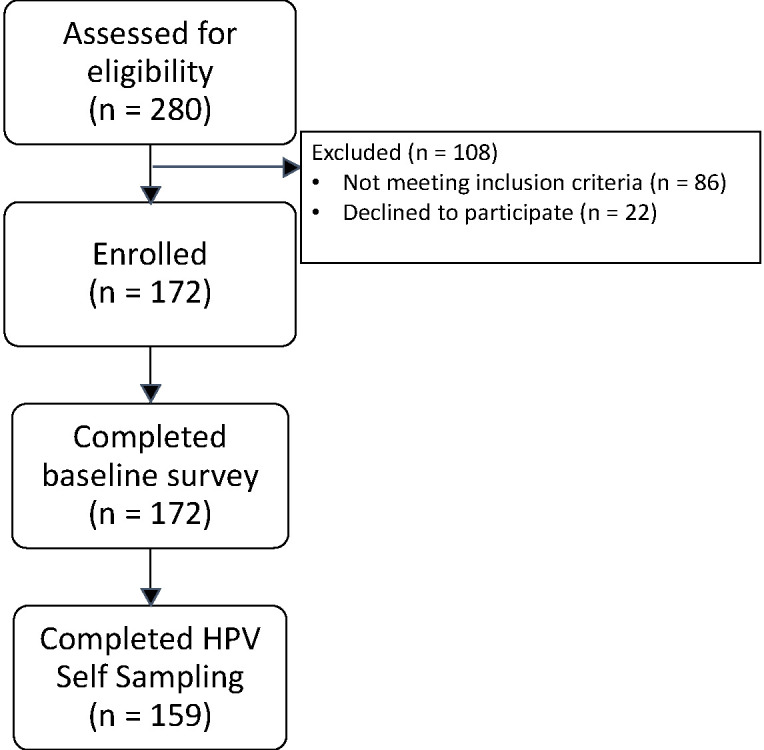
Flow diagram for patient recruitment, enrollment, and participation in the study.

Women aged 25- 65 years of age who were enrolled in the parent study were recruited to participate in the hrHPV pilot study. Attendees were recruited with a coordinated series of media and community outreach campaigns, including announcements delivered over the radio and from a mobile truck, banners displayed in public places, and leaflets distributed at church services. Community-wide sensitization meetings were held to provide information about the VIA screening and embedded hrHPV study and to answer questions. Eligible women were identified during these community meetings or at the time of the health fair and were referred to trained research assistants for a detailed explanation of the study and to obtain written informed consent. VIA was offered to eligible attendees independent of study participation. Women were excluded if they were (1) pregnant; (2) had a known gynecologic tract malignancy; or (3) had a prior hysterectomy (**Figure 1**).

During the health fair, all women received general education on cervical cancer prevention, which was adapted from the Alliance for Cervical Cancer Prevention “Planning and Implementing Cervical Cancer Prevention and Control” programmatic guide [13]. Structured interviews were used to gather information on past medical, reproductive, and sexual history, including prior cervical cancer preventive care. A literacy test was performed to assess study participants’ ability to read short message service (SMS) text messaging. Cellular telephone contact information was obtained from each participant. Participants were then directed to the screening tents, where they received instructions for self-sampling and then collected the HPV sample. VIA screening was then performed by trained nurses and physicians, followed by cryotherapy for women meeting treatment criteria by VIA per WHO guidelines. Screen-positive women are eligible for cryotherapy if the entire lesion is visible, the squamocolumnar junction is visible, and the lesion does not cover more than 75% of the ectocervix [19].

Following screening with hrHPV self-sampling and VIA, participants were administered a post-screen survey to assess their experiences, including their overall impression, level of comfort, pain, embarrassment, and confidence associated with each screening test. Participants were provided contact information for the Cervical Cancer Prevention (CCP) clinic at Mbarara Regional Referral Hospital (MRRH). Women who screened positive or suspicious for cancer with VIA, or who had received cryotherapy, were instructed to present to the CCP for clinical follow-up.

All samples were transported to a central laboratory at the MRRH. All HPV samples were then analyzed using GeneXpert Xpert® HPV (Cepheid, Sunnyvale, Calif.), which reports results as HPV 16, HPV 18/45, other hrHPV (31, 33, 35, 52, 58; 51, 59; 39, 56, 66, 68), HPV negative, or invalid. Tests returning with invalid results were tested in triplicate.

Test results were delivered to women by SMS using an automated web-based algorithm developed by Innovation Streams Limited (Mbarara, Uganda) (Appendix S1 in the **Online Supplementary Document**). Pre-defined messages were scripted in Runyankore for women with HPV positive results and women with HPV negative results. Women who were HPV positive were instructed to follow-up at the CCP at MRRH. Women who were HPV negative were instructed to repeat cervical cancer screening in 5 years (1 year for women with HIV). Women who had a suspicious finding based on VIA were instructed to return at the health fair, and again through SMS instructions for follow-up, independent of HPV result, but were informed of their HPV test results.

Once test results were available, an automated SMS containing the scripted content was sent to the participant’s phone number at no charge to the recipient. SMS text messages were sent monthly for a period of six months following the health fair. After six months, all participants received a follow-up phone survey, which we conducted in order to ascertain whether they had received and/or understood the SMS message, and/or to elicit their reasons for non-follow up (ie, for those participants who were either HPV positive or VIA positive and had not yet presented for care).

During the study period and following the health fair, nurses at the CCP kept a log of all returning participants. Participants who returned for follow-up underwent colposcopy, biopsy, and recommended treatment under the discretion of the clinic physician and nurses, and per WHO and Uganda cervical prevention guidelines [20]. Clinical chart abstraction to ascertain clinical care was performed for participants returning to clinic.

We aimed to understand the effectiveness of this intervention to achieve screening goals as well as to understand implementation outcomes for each component of the intervention. Thus we evaluated this pilot on both implementation outcomes (penetration, appropriateness, acceptability and feasibility) and service outcomes (effectiveness) chosen from Proctor’s model of implementation research [21]. *Penetration* was defined as the percentage of women meeting eligibility criteria for screening who consented to participate and had sample collection performed. *Appropriateness* was defined as the percentage of women with the literacy skills to read results via SMS and who had a phone to receive SMS results. *Acceptability* was measured using mean scores from the post-screen survey. *Feasibility* was defined as the percentage of women obtaining samples, having valid test results, and receiving SMS results. *Effectiveness* was defined as the number of women with positive results returning for follow-up in the CCP. Stata statistical software (version 16.1, College Station, TX, USA) was used for analyses.

This study was approved by the Institutional Review Boards at Mbarara University of Science and Technology, Mbarara Uganda, and Massachusetts General Hospital, Boston, MA. Study data were collected and managed using REDCap electronic data capture tools hosted at Partners Healthcare [22].

## RESULTS

Over a 5-day period, 280 women were screened. Of these, 194 (69%) met inclusion criteria, of whom 172/194 (89%) were enrolled and completed the baseline survey and 159/194 (82%) collected HPV self-samples, achieving penetrance of 79% of the intended population (**Figure 1**). Participating women had a mean age of 41.2 years (range, 25-63 years). Most women were married (120 [76%]), had 2 or fewer lifetime partners (111 [71%]), and had a mean age of first intercourse of 21.2 years (range, 14-32 years) (**Table 1**).

**Table 1:** Summary characteristics of women who underwent HPV Self sampling (n = 159)

Characteristics	N (%)
**Mean age in years (range)**	41.2 (25-63)
**Literacy**	126 (79)
**Relationship status:**	
Married	120 (76)
Partnered	1 (0.6)
Widowed	19 (12)
Single	6 (4)
Divorced	13 (8)
Mean age at first intercourse in years (range)	21.2 (14-32)
**Number of lifetime partners:**	
0-2	111 (71)
3-4	34 (22)
5-6	11 (7.0)
≥7	1 (0.6)
**Mean number previous pregnancies** ± **SD (range)**	5.5 ± 0.49 (0-12)
**Mean number live births** ± **SD (range)**	4.5 ± 0.17 (0-10)
**Postmenopausal**	46 (29)
**Cervical cancer screening history:**	
No prior screening	102 (65)
**Previous screening method:**	
VIA	37 (66)
Pap	6 (11)
HPV	9 (16)
Unsure	4 (7)
**Prior screen results:**	
Normal	51 (93)
Abnormal	1 (2)
Unsure	3 (5)

HPV – human papillomavirus, SD – standard deviation, VIA – visual inspection after acetic acid application

Among those who participated in hrHPV self-sampling, 56/159 (35%) women had undergone previous screening. VIA was the most commonly reported modality (37/56 [66%]), and most women reported that their prior results (51/56 [91%]) were reported to them as normal. (**Table 1**).

### VIA and HPV Outcomes

VIA, HPV, SMS text, and follow-up outcomes are listed in **Table 2**. Adequate VIA screening was achieved for 138 (87%) women enrolled. Fourteen (10%) were VIA positive, 114 (83%) were VIA negative, and 8 (6%) had an inconclusive or unsatisfactory result. Of the women who were VIA positive, nine (6%) received cryotherapy treatment on site, and one (1%) was referred to a higher level of care for a clinical impression of invasive cancer.

**Table 2:** SMS text interpretation and follow-up among HPV positive and HPV negative women (N, %)

Outcomes	Overall (n = 159)	HPV positive (n = 27)
VIA screening results:		
Total screened with VIA	138 (87)	27 (100)
VIA negative	114 (83)	18 (67)
VIA positive	14 (10)	4 (15)
Suspicious for cancer	1 (1)	0
Unsatisfactory VIA	8 (6)	1 (4)
Cryotherapy	9 (6)	3 (11)
HPV test result:		
Positive	27 (17)	–
Negative	129 (81)	–
Invalid	4 (2.5)	–
HPV genotype:		
16	–	6 (20)
18/45	–	6 (20)
Other high risk	–	21 (78)
Co-infection with one or more hrHPV strain		5 (19)
SMS text delivery:		
Receipt of SMS text messages		
Report receiving SMS text	74 (46.2)	17 (63)
Report not receiving SMS text	49 (30.6)	7 (26)
Unsure if received SMS text	16 (10.0)	2 (7)
Receipt of clinical follow-up:		
Presented for clinical follow up	9 (6)	6 (22)
Type of clinical follow up		
Pap	0 (0)	0 (0)
Colposcopy	0 (0)	2 (7)
Cryotherapy	9 (6)	4 (15)
Referral for surgery or palliation	0 (0)	0 (0)

HPV – human papillomavirus, VIA – visual inspection after acetic acid application

Of the 159 women who obtained HPV self-samples, 27 (17%) were hrHPV positive, 120 (81%) were hrHPV negative, and 4 (3%) had an invalid result. Of the 27 women with hrHPV positive results, 6/27 (20%) were positive for HPV 16, 6/27 (20%) were positive for HPV 18 or 45, and 21/27 (78%) were positive for other high-risk HPV types. Co-infection with one or more high risk strain was present in five (19%) women.

Among those with positive hrHPV results, 4 (17%) also had positive findings on VIA, although none were suspicious for cancer. Three (11%) underwent cryotherapy. HPV outcomes according to VIA results are included in **Table 2**.

### SMS Text Messages

**Figure 2:**
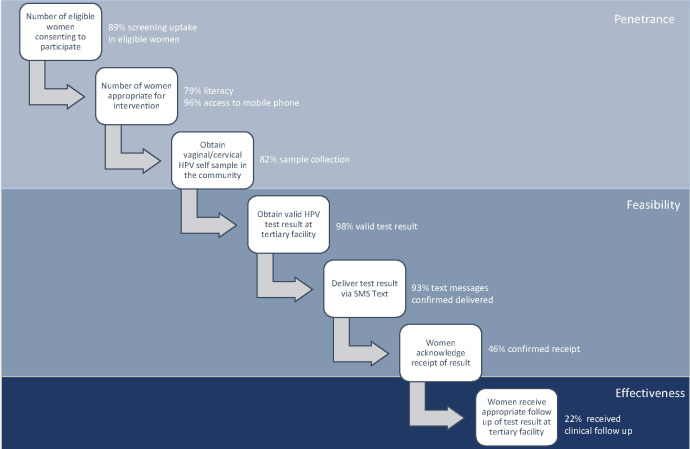
Nodes for effective linkage to care using community-based HPV self-sampling with tertiary hospital referral and outcomes. The flow diagram demonstrates key points for intervention and linkage for effectiveness in the cervical cancer screening cascade.

Nearly all (153 [96%]) participants who obtained HPV self-samples provided a mobile phone number (15 of whom provided a smartphone). Most (126 [79%]) participants were able to demonstrate literacy on enrollment, by completing enrollment surveys and reading alound SMS text messages options. SMS text messages were confirmed as delivered to 147 (93%) participants (**Figure 2**). Six messages could not be delivered, none of which were intended for women with HPV positive results. Most women (139 [78%]) were reachable by phone at the 6-month endpoint after SMS messages were sent. Of these, about half (74/139 [53%]) reported receipt of messages, and 49 (50%) denied receiving messages; 20 (13%) could not be reached by phone (**Table 2**).

Of the 27 women with a hrHPV positive results, all of whom were sent SMS messages instructions to follow-up, 6 (22%) followed up at the CCP for further screening, following receipt of text messages. Three women (11%) received cryotherapy, 2 (4%) had colposcopy and 1 (4%) had a pap smear. All 27 women with hrHPV positive results were reached by phone at the six-month mark. The 21 women who had not yet presented for follow-up screening reported the following reasons: lack of transportation (n = 11), disbelief of results (n = 5), lack of childcare (n = 4), asymptomatic (n = 3), forgot (n = 3), avoidance of male provider and/or pelvic exam (n = 2), lack of trust in care providers (n = 1), perception of already receiving treatment at health fair (n = 1).

### Acceptability

Most women rated self-sampling highly and overall reported less embarrassment, less pain, higher confidence, and higher ease of screening with self-collected HPV compared with VIA screening (**Table 3**). The median differences, when compared to the standard deviation values, suggest large effect sizes, ranging from 0.6-0.7 standard deviation units for “confidence that screen performed correctly” to >1 standard deviation unit for “pain experienced during screening” and “likelihood to recommend screening” (**Table 3**). Individual Likert scale responses are included in Appendix S2 of the **Online Supplementary Document**.

**Table 3:** Quality of self-collected HPV (median, SD) compared to visual inspection with acetic acid cervical cancer screening scale specific responses (n = 159)

	Median (SD)			
	**Self-collected**	**VIA**	**Effect size**	*P-*value*
Embarrassment felt during screening (1 = very embarrassed, 5 = not at all embarrassed)	4.8 (0.8)	3.6 (1.5)	0.8-1.5	<0.001
Pain felt during screening (1 = severe discomfort, 5 = no discomfort)	4.3 (1.2)	2.7 (1.4)	1.1-1.3	<0.001
Confidence that screen performed correctly (1 = not at all confident, 5 = very confident)	4.8 (0.6)	4.4 (0.7)	0.6-0.7	<0.001
Ease of performing screening during health fair (1 = not that easy, 5 = very easy)	4.6 (0.6)	3.8 (1.1)	0.9-1.5	<0.001
Likelihood to recommend screening (1 = very unlikely, 5 = highly likely)	4.7 (0.6)	0.9 (1.0)	6.3-5.2	<0.001

HPV – human papillomavirus, SD – standard deviation, VIA – visual inspection after acetic acid application

*Wilcoxon rank test used to compare responses.

## DISCUSSION

In this study we piloted the implementation of community-based HPV self-sampling for primary cervical cancer screening in a rural agrarian-based population in Uganda, using SMS text messaging to notify study participants of their results. We achieved a high penetrance of eligible women attending the health fair (79%). Most women were appropriate for the intervention, having demonstrated high rates of literacy (79%) and access to a mobile phone (96%). We demonstrated high feasibility in obtaining self-collected HPV samples, obtaining valid test results (98%), and confirming delivery of SMS texts (93%). However, result receipt by SMS was only confirmed as successful in about 50% of women who were reachable by phone at the 6-month endpoint, with only 22% of women with hrHPV positive results following up at the tertiary care facility (**Figure 2**).

Our evaluation approach using the Proctor model enabled us to identify target areas in the cascade of cervical cancer screening for improvement of implementation. We demonstrated that self-sample collection can be performed with high penetrance, and that the context of the community is appropriate for an intervention based on SMS text result notification. However, there was high non-follow up rate in our participants. More work is needed to understand this finding, especially since SMS has been tested and demonstrated to successfully improve linkage to care among people living with HIV in the same catchment area as our study [15,23]. However in this study, this strategy for linkage to care was tested among people who had already been linked to HIV care at MRRH, participation in the study was restricted to those with confirmed cell phone ownership, and SMS messaging was combined with transportation reimbursement. In our study, participants may not have had engagement with, or previously visited, MRRH and were not provided funds for transportation to the clinic. Indeed, difficulty with transportation was cited as the most common reason for lack of follow-up. These findings demonstrate that SMS messaging may not fulfill the promise of improving linkage to care, and that other implementations gaps must be addressed in the cervical cancer screening cascade.

Primary prevention of cervical cancer hinges on successful vaccination. Currently available vaccines target HPV 16 and -18 genotypes [24]. In our cohort, although hrHPV prevalence was 21%, HPV 16 prevalence was 2.7% and HPV 18/45 prevalence was 1.9%, which is similar to published findings from a large community based study in western Uganda [25]. This may have implications for the efficacy of vaccination in Uganda, and underscores the need for effective secondary prevention through screening.

Our findings should be interpreted in light of several limitations. Our cohort was limited to women who presented to the community health fair and who were willing to participate in cervical cancer screening,. Due to small sample size, we were unable to assess for predictors for follow-up. There was no control group to facilitate comparison in follow-up in those who did vs did not receive SMS follow-up and limited our ability to analyze the efficacy of text message follow-up. There was a 6-month interval from screening to result notification, which may have decreased the willingness of participants to engage in follow-up. This was done according to study protocol, given that the progression from infection to cancer occurs over decades, and given that all women who completed sampling had undergone a physical exam during VIA screening by experienced gynecologic oncology nurses and physicians; This was done according to study protocol, given that the progression from infection to cancer occurs over decades, and given that all women who completed sampling had undergone a physical exam during VIA screening by experienced gynecologic oncology nurses and physicians. It is also possible that women presented for follow-up care at health facilities other than MRRH, causing us to underestimate the rate of appropriate follow-up in this cohort. Finally, HPV self-sampling was performed concurrently with VIA screening at the time of the health fair. This was done to achieve a service delivery goal of cervical cancer screening using the current standard of care available. However, our simultaneous use of both methods of screening may have reduced willingness to follow-up among women receiving HPV positive results due to their perception of having already being screened.

Despite these limitations, our study presents a novel approach to community-based primary screening for cervical cancer and identifies key areas in the cervical cancer screening cascade and linkage to care that can be targeted for improvement. Though small in number, the willingness of women and reliance on community health workers for result delivery, unprompted by the study, reveals another potential avenue to achieve community-based screening, which may be promising. Further research will be need needed to examine the acceptability and performance of a community health worker-based HPV self-sampling strategy for community-based cervical cancer screening. This, coupled with strategies to reduce or cover transportation costs, either by providing travel reimbursements, or enabling follow-up screening at health facilities closer to women’s homes, may improve success rates across the cascade of care.

## Additional material


Online Supplementary Document
