# Supplementary material for: Implementing community-based human papillomavirus self-sampling with SMS text follow-up for cervical cancer screening in rural, southwestern Uganda
Source: J Glob Health. 2021 Dec 25;11:04036. doi: 10.7189/jogh.11.04036 (PMC8709902; doi:10.7189/jogh.11.04036)
Supplement: Online Supplementary Document [file jogh-11-04036-s001.pdf]

**Appendix 1.** Block Algorithm for the Text Messages Based on HPV Status, VIA Status, and Receipt of Treatment.

| | HPV POSITIVE | HPV NEGATIVE |
|---|---|---|
| VIA POSITIVE, *untreated* | SMS:<br>"Your cancer screening test was abnormal. Please come to the Cervical Cancer Prevention Clinic at MRRH as soon as possible for follow-up." | SMS:<br>1. "Your screening test was normal. You do not need any follow-up now, but may need repeat screening in 1 -5 years."<br>2. "Please remember to keep your appointment at the Cervical Cancer Prevention Clinic at MRRH as soon as possible for follow-up." |
| VIA POSITIVE, *treated* | SMS:<br>1. "Your cancer screening test was abnormal. Please come to the Cervical Cancer Prevention Clinic at MRRH as soon as possible for follow-up."<br>2. "Please remember to keep your appointment at the Cervical Cancer Prevention Clinic at MRRH as soon as possible for follow-up." | SMS:<br>1. "Your screening test was normal. You do not need any follow-up now, but may need repeat screening in 1 -5 years."<br>2. "Please remember to keep your appointment at the Cervical Cancer Prevention Clinic at MRRH as soon as possible for follow-up." |
| VIA NEGATIVE | SMS: "Your cancer screening test was abnormal. Please come to the Cervical Cancer Prevention Clinic at MRRH as soon as possible for follow-up. | SMS: "Your screening test was normal. You do not need any follow-up now, but may need repeat screening in 1 -5 years." |

**Appendix 2.** Structured Post Screening Assessment Comparing Screening with VIA vs. Self-Collected HPV Swabs.

### A. Experience with Self Sampling (%)

| | | Response | | | | |
|---|---|---|---|---|---|---|
| | | 1 | 2 | 3 | 4 | 5 |
| | N | n (%) | n (%) | n (%) | n (%) | n (%) |
| Embarrassment felt during screening (1=very embarrassed, 5=not at all embarrassed) | 156 | 5 (3.21) | 2 (1.28) | 2 (1.28) | 6 (3.85) | 141 (90.4) |
| Pain felt during screening (1=severe discomfort, 5=no discomfort) | 155 | 9 (5.81) | 10 (6.45) | 14 (9.03) | 28 (18.1) | 94 (60.7) |
| Confidence that screen performed correctly (1=not at all confident, 5=very confident) | 153 | 1 (0.65) | 2 (1.31) | 2 (1.31) | 12 (7.84) | 136 (88. 9) |
| Ease of performing screening during health fair (1=not that easy, 5=very easy) | 156 | 0 | 2 (1.32) | 8 (5.13) | 34 (21.8) | 112 (71.8) |
| Likelihood to recommend screening (1=very unlikely, 5=highly likely) | 156 | 1 (0.64) | 1 (0.64) | 2 (1.28) | 29 (18.6) | 123 (78.9) |

### B. Experience with VIA (%)

| | | Response | | | | |
|---|---|---|---|---|---|---|
| | | 1 | 2 | 3 | 4 | 5 |
| | N | n (%) | n (%) | n (%) | n (%) | n (%) |
| Embarrassment felt during screening (1=very embarrassed, 5=not at all embarrassed) | 151 | 23 (15.2) | 23 (15.2) | 16 (10.6) | 26 (17.2) | 63 (41.7) |
| Pain felt during screening (1=severe discomfort, 5=no discomfort) | 149 | 31 (20.8) | 30 (20.1) | 33 (22.2) | 32 (21.5) | 23 (15.4) |
| Confidence that screen performed correctly (1=not at all confident, 5=very confident) | 146 | 1 (0.68) | 1 (0.68) | 11 (7.53) | 61 (41.8) | 72 (49.3) |
| Ease of performing screening during health fair (1=not that easy, 5=very easy) | 149 | 8 (5.37) | 7 (6.04) | 27 (18.1) | 65 (43.6) | 42 (28.2) |
| Likelihood to recommend screening (1=very unlikely, 5=highly likely) | 151 | 8 (5.30) | 5 (3.31) | 7 (4.64) | 67 (44.4) | 64 (42.4) |
